# Recent advances in cancer immunotherapy

**DOI:** 10.1186/s13045-017-0460-9

**Published:** 2017-04-24

**Authors:** Weijing Sun

**Affiliations:** University of Pittsburgh Cancer Institute, 5150 Centre Avenue, Fifth Floor, Pittsburgh, PA 15232 USA

Laboratory and clinical investigations of immunotherapy in cancer treatment have been underway for the past several decades by attempting to stimulate, enhance, and modulate the immune system to detect and destroy cancer cells. Although immunotherapy has shown success in a wide variety of cancers previously: BCG in superficial bladder cancer, interferon-alpha in melanoma, and high-dose interleukin in renal cell carcinoma, allogeneic stem cell transplantation in hematologic malignancies, et al., the benefits are only limited to a small population of patients, and mechanisms are less clear.

With the new advances in molecular and tumor biology, new development of technology in “-omics” and data analysis, there have been dramatic successes in the cancer immunotherapy recently. Targeting the immune checkpoint pathways to activate the host immune system against cancer cells is one such novel approach that is rapidly evolving in the recent years. Anti-CTLA4 and anti-PD1 monoclonal antibodies have taken us forward into the realm of longer survival and durable responses with the possibility of cure in a continuously increasing proportion of patients. Combination immunotherapeutic strategies and novel immunotherapeutic agents are being tested at an accelerated pace where the outlook for long-term survival benefits for the majority of patients appears brighter than ever. With the FDA’s approval of anti-CTLA-4 and PD-1/PD-L1 agents, and encouraging research data of combination of immune checkpoint inhibitors and success of CAR-T in hematologic malignances, revolutionary changes of cancer therapy will be soon to come.

In this mini-review series, authors have summarized the development of immunotherapy in the field of melanoma, lung cancer, GU cancers, GI cancers, and hematological malignances. Mechanisms of different immunotherapeutic approaches were detailed. Some important issues in immunotherapy were discussed as well, e.g., identification of clinically relevant predictive and prognostic biomarkers to help define subgroups of patients who are most likely to benefit from various immunotherapies; management of immunotherapy-related toxicities and resistance; and future directions and approaches.

